# Supplementary material for: Biological Characterization and Evaluation of the Therapeutic Value of Vibrio Phages 4141 and MJW Isolated from Clinical and Sewage Water Samples of Kolkata
Source: Viruses. 2024 Nov 6;16(11):1741. doi: 10.3390/v16111741 (PMC11598976; doi:10.3390/v16111741)
Supplement: Supplementary file 1 [file viruses-16-01741-s001.zip › viruses-3286314-supplementary-figure - to XML.pdf]

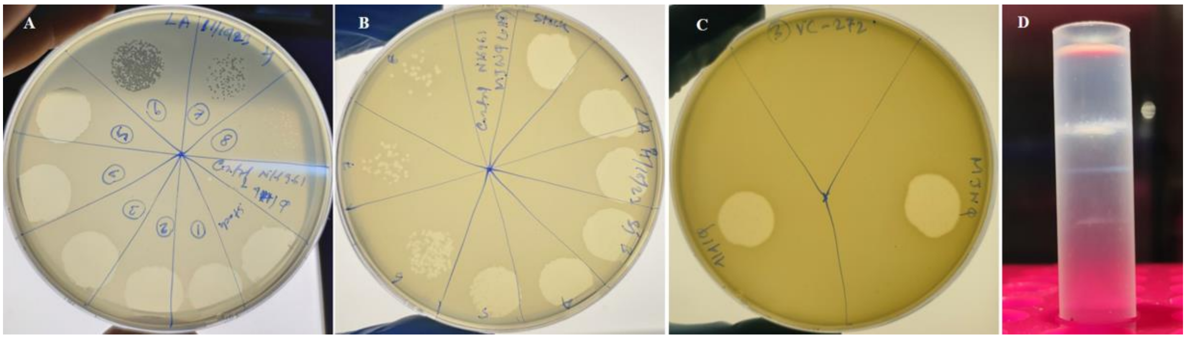

Figure S1: Routine test dilution test: (A) Vibrio phage 4141; (B) Vibrio phage MJW; (C) phage sensitivity with minimum inhibitory concentration (MIC) on clinical samples; and (D) Cs-Cl gradient purification image, and blue band indicated pure phage particles. The purified phage stock solution was serially diluted to make 102 PFU/mL to 1010 PFU/mL, and each dilution was tested to evaluate MIC.

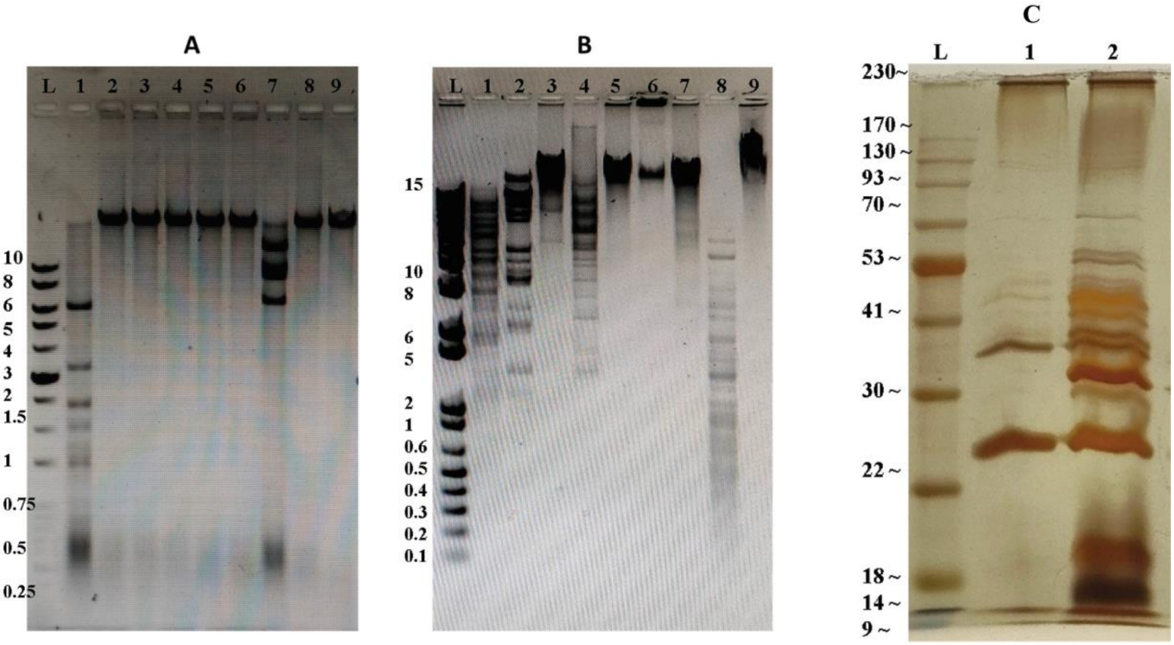

Figure S2: 1% Agarose gel electrophoresis and SDS PAGE analysis of the 4141 phage and the MJW phage: L-1Kb DNA ladder (A) Vibrio phage 4141 digested with 1.HindIII; 2.PstI; 3.BamHI; 4.EcoRI; 5.XbaI; 6.ApaI; 7.NdeI; 8.DpnI; and 9.Uncut DNA. (B) L-1Kb+ DNA ladder Vibrio Phage MJW digested with 1.HindIII; 2. PstI; 3. BamHI; 4. EcoRI; 5. XbaI; 6. ApaI; 7. NdeI; 8. DpnI; and 9. Uncut DNA. (C) Ladder-L: Major structural protein of 1. Vibrio phage 4141 and 2. Vibrio phage MJW was analyzed by 12% SDS PAGE. Silver staining was performed for structural protein identification.
