# Supplementary material for: Biological Characterization and Evaluation of the Therapeutic Value of Vibrio Phages 4141 and MJW Isolated from Clinical and Sewage Water Samples of Kolkata
Source: Viruses. 2024 Nov 6;16(11):1741. doi: 10.3390/v16111741 (PMC11598976; doi:10.3390/v16111741)
Supplement: Supplementary file 1 [file viruses-3286314-supplementary-VipTree S3-to XML.pdf › viruses-3286314-supplementary-VipTree S3-to XML.pdf]

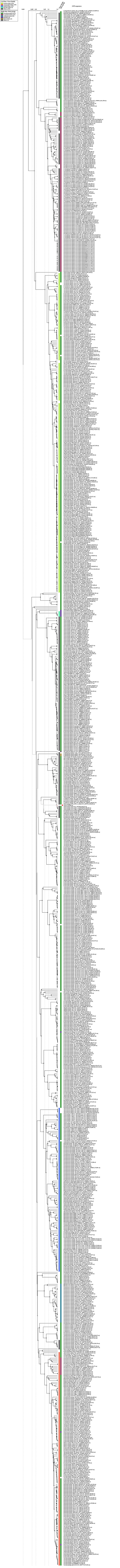
Left line: Virus family
Straboviridae (234)
Autographiviridae (159)
Schitoviridae (122)
Herelleviridae (4)
Others (329)
Right line: Host group
Pseudomonadota (972)
Cyanobacteriota (131)
Actinomycetota (29)
Bacillota (8)
Bacteroidota (2)
Others (1)
Virus family
Host group
1159 sequences
0.001
0.005
0.01
0.05
0.1
0.5
