# Supplementary material for: Biological Characterization and Evaluation of the Therapeutic Value of Vibrio Phages 4141 and MJW Isolated from Clinical and Sewage Water Samples of Kolkata
Source: Viruses. 2024 Nov 6;16(11):1741. doi: 10.3390/v16111741 (PMC11598976; doi:10.3390/v16111741)
Supplement: Supplementary file 1 [file viruses-3286314-supplementary-VipTree S2-to XML.pdf › viruses-3286314-supplementary-VipTree S2-to XML.pdf]

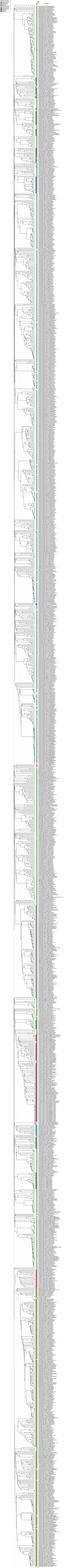
Left line: Virus family
Autographiviridae (382)
Drexlerviridae (120)
Schitoviridae (17)
Straboviridae (10)
Others (260)
Right line: Host group
Pseudomonadota (1082)
Actinomycetota (747)
Cyanobacteriota (147)
Bacillota (5)
Bacteroidota (1)
Others (11)
Virus family
Host group
2039 sequences
